# Atopic Dermatitis in Children Under 5: Prevalence Trends in Central, Eastern, and Western Europe

**DOI:** 10.3390/children10081275

**Published:** 2023-07-25

**Authors:** Irena Ilic, Andjelka Stojkovic, Vesna Velickovic, Ivana Zivanovic Macuzic, Milena Ilic

**Affiliations:** 1 Faculty of Medicine, University of Belgrade, 11000 Belgrade, Serbia; 2 Department of Pediatrics, Faculty of Medical Sciences, University of Kragujevac, 34000 Kragujevac, Serbia; andja410@mts.rs; 3 Clinic of Pediatrics, University Clinical Center Kragujevac, 34000 Kragujevac, Serbia; vesna.velickovic@mts.rs; 4 Department of Anatomy, Faculty of Medical Sciences, University of Kragujevac, 34000 Kragujevac, Serbia; ivanaanatom@yahoo.com; 5 Department of Epidemiology, Faculty of Medical Sciences, University of Kragujevac, 34000 Kragujevac, Serbia; drmilenailic@yahoo.com

**Keywords:** atopic dermatitis, children under 5, prevalence, trend, Joinpoint regression analysis

## Abstract

Background: Atopic dermatitis is a major public health issue worldwide, but data on trends in prevalence in children in European countries are sparse. The aim of this study was to assess the trends in the prevalence of atopic dermatitis in children under 5 in the Central, Eastern, and Western European sub-regions from 1990 to 2019. Methods: In this study, a descriptive, observational epidemiological method was applied. In addition, an ecological study design was used. Joinpoint regression analysis was used to assess trends. Results: A total of 118 million (54 million males and 64 million females) prevalent cases of atopic dermatitis in children under 5 were reported in European countries in 1990–2019. More than half of all cases of atopic dermatitis in children under 5 in Europe were recorded in the Western European sub-region. The highest prevalence rates of atopic dermatitis in children under 5 were observed in the Eastern European sub-region, with the highest rates in both males and females recorded in Estonia (equally at about 15,000 per 100,000), followed by the Russian Federation (equally at about 12,000 per 100,000). Moreover, the lowest prevalence rates were reported in the Eastern European sub-region (equally at about 5000 per 100,000 in Romania and Latvia). A sex disparity in the prevalence and trends of atopic dermatitis in children under 5 was observed. A significantly increased trend in atopic dermatitis prevalence in children under 5 was recorded in the Eastern European sub-region from 1990 to 2019 (by +0.3% per year in males and by +0.1% per year in females). Conversely, in the Western and Central European sub-regions, trends in the prevalence of atopic dermatitis in children under 5 slightly decreased (about −0.1% per year). The Pearson coefficient showed a significant negative correlation between the prevalence of atopic dermatitis in children under 5 and the Human Development Index in most countries in the Eastern European sub-region, while a significant positive correlation was reported between the prevalence and HDI in high-income countries in the Western European sub-region. Conclusions: High prevalence rates and mostly stable trends during the last three decades make atopic dermatitis still a major health issue in children under 5 in European countries.

## 1. Introduction

Atopic dermatitis represents a chronic inflammatory skin condition that presents with eczematous skin lesions and dry, itchy, and painful skin [1]. Globally, reports on the burden of atopic dermatitis show significant variations worldwide, owing to differences in disease definition, method of diagnosis, and study methodology [2]. A large systematic review of 378 cross-sectional and cohort studies that was conducted worldwide and comprised several million persons revealed that the reported 1-year prevalence of atopic dermatitis in children ranged from 0.94% to 22.6%, while, in adults, the 1-year prevalence varied between 1.2% and 17.1% [2]. Despite global age-standardized prevalence rates being stable over the 1990–2017 period, many regions experienced a significant increase in age-standardized prevalence rates over this period, including the High-income region, Southeast Asia, East Asia, and Oceania; Latin America and the Caribbean; and North Africa and the Middle East [3]. Further, most studies that have assessed the atopic dermatitis incidence and prevalence in Europe have reported an increase in burden, albeit not all [2].

For the majority of affected persons, disease onset happens in early childhood; however, there is increasing evidence for adult-onset atopic dermatitis [3,4,5,6,7,8]. Atopic dermatitis represents a complex public health issue because it has a variety of clinical presentations (these phenotypes include acute and chronic forms, intrinsic and extrinsic, and variants differing by morphology and localization), which might make diagnosis and treatment difficult [9]. There have been significant improvements in the therapeutic modalities for atopic dermatitis, involving a multidisciplinary approach, to which the results of international and multicentric studies of the use of therapeutics (e.g., monoclonal antibodies such as dupilumab or Janus kinase inhibitors such as upadacitinib) point [10,11]. Therefore, understanding the temporal and geographic trends of atopic dermatitis should help in managing the disease burden. Globally, research shows that the prevalence of atopic dermatitis is the highest in children up to the age of 5; it then decreases in young adults and again rises in persons of middle age and older [3,7]. Estimates of the differences in atopic dermatitis prevalence by sex are not consistent across studies. While the 1-year prevalence and lifetime prevalence of atopic dermatitis diagnosed by a physician were higher in females, in children, the point prevalence was higher in males up to the age of 1 year. For adults, the point prevalence was higher in females [2]. Research shows that, globally, atopic dermatitis has the highest age-standardized disability-adjusted life years (DALYs) rate among all skin diseases and is ranked 15th in age-standardized DALY rates among non-fatal diseases. In Europe, the Western European region was among the five regions in the world that had the highest age-standardized DALY rates of atopic dermatitis, while Central Europe and Eastern Europe were among the five regions of the world with the lowest rates [3]. Atopic dermatitis is associated with a higher risk of autoimmune disorders, mental illnesses, sleeping disorders, and cardiovascular diseases, and it also affects negatively the quality of life of affected persons [5]. Many studies have shown that atopic dermatitis leads to significant direct and indirect costs, both for patients and their families [3,4,5]. In order for the key stakeholders and healthcare professionals to effectively plan healthcare resources and their allocation, it is important to elucidate the disease burden of atopic dermatitis. Atopic dermatitis is a major public health issue worldwide, but data on the trends of prevalence in children in European countries are sparse. The aim of this research was to assess the trends in the prevalence of atopic dermatitis in children under 5 in European countries.

## 2. Methods

The trends in the prevalence of atopic dermatitis in children under 5 in the Central, Eastern, and Western European sub-regions from 1990 to 2019 were evaluated using the latest data of the Global Burden of Disease (GBD) 2019 study [8].

### 2.1. Study Design

This research applied a descriptive, observational epidemiological method. In addition, an ecological study design was used.

### 2.2. Study Population

Based on the GBD 2019 study, the world is divided into 21 regions according to epidemiological similarities and geographical proximity: the Central European sub-region includes 13 countries (Albania, Bosnia and Herzegovina, Bulgaria, Croatia, Czechia, Hungary, North Macedonia, Montenegro, Poland, Romania, Serbia, Slovakia, and Slovenia), the Eastern European sub-region includes 7 countries (Belarus, Estonia, Latvia, Lithuania, Republic of Moldova, Russian Federation, and Ukraine), and the Western European sub-region includes 24 countries (Andorra, Austria, Belgium, Cyprus, Denmark, Finland, France, Germany, Greece, Iceland, Ireland, Israel, Italy, Luxembourg, Malta, Monaco, Netherlands, Norway, Portugal, San Marino, Spain, Sweden, Switzerland, and United Kingdom), comprising about 745 million inhabitants, including nearly 35 million children under 5 [8].

### 2.3. Data Source

Annual numbers of cases of atopic dermatitis in children under 5 from 1990 to 2019, by year, sex, and country, were extracted from the GBD 2019 study [8]. GBD 2019 provides a comprehensive assessment of the all-cause and cause-specific burden for 369 diseases and injuries and 87 risk factors across the world from 1990 to 2019. GBD 2019 uses all available disease and injury data from a range of data source types, including vital statistics, civil registration, household surveys, registries, and hospital records. Based on the International Classification of Diseases, atopic dermatitis is defined as code L20 (L20.0: Besnier prurigo; L20.8: Other atopic dermatitis; L20.9: Atopic dermatitis, unspecified). The estimates from the GBD study follow the Guidelines for Accurate and Transparent Health Estimates Reporting (GATHER) [12].

### 2.4. Measures

Age- and sex-specific rates for the prevalence of atopic dermatitis in children under 5 were presented. All rates were reported per 100,000 persons. In this study, prevalence rates by 3 sub-regions and 44 countries were determined. The analyses were performed for males and females separately.

The Human Development Index (HDI) represents a composite measure that is used to determine the development of a country. The HDI represents average achievement in three key dimensions: a long and healthy life (life expectancy at birth), knowledge (mean of years of schooling for adults aged 25 years and over), and a decent standard of living (gross national income per capita) [13]. The United Nations Development Programme developed the HDI, and, based on the value of the HDI (which ranges from 0 to 1, whereby 1 is considered ideal), countries score a higher level of HDI when the life expectancy is longer, the level of education is high, and the gross national income per capita is high.

### 2.5. Statistical Analysis

Temporal trends for atopic dermatitis prevalence rates were described via Joinpoint regression analysis (Joinpoint regression software, Version 4.9.0.0; National Cancer Institute, Bethesda, Maryland, USA—March 2021, available through the Surveillance Research Program of the US National Cancer Institute) [14].

The Joinpoint regression program was used to find the best-fit line among a series of joined straight lines on a log scale in the annual age-specific rates, with the calendar year as the regression variable. The test of significance used a Monte Carlo Permutation method. The grid search method was used [15]. In our analysis, one segment line was described for each model. The Average Annual Percent Change (AAPC) with its corresponding 95% confidence interval was used to describe the trend, as a summary measure of the trend [16].

When a trend’s slope was statistically significant (*p* < 0.05, based on the statistical significance of the AAPC compared to zero), the terms “significantly increasing trend” or “significantly decreasing trend” were used. When the AAPC was equal to zero, the trend in prevalence rates was neither increasing or decreasing. When an AAPC (with its 95% CI) included zero, the term “non significant trends” (*p* > 0.05) was used, either as “increase” (for AAPC > 0.5%) or “decrease” (for AAPC < −0.5%). Moreover, the term “stable” was used for AAPC between −0.5% and 0.5%.

The correlation between the prevalence rates of atopic dermatitis and HDI was assessed using the Pearson correlation coefficient. Statistical analyses were conducted using the SPSS software (Version 20.0, Chicago, IL, USA). A *p* value < 0.05 indicated statistical significance for all tests.

### 2.6. Ethics Statement

This study was approved by the Ethics Committee of the Faculty of Medical Sciences, University of Kragujevac (No. 01–14321). The study was conducted using publicly available data, and the data were fully aggregated and anonymized.

## 3. Results

**Figure 1:**
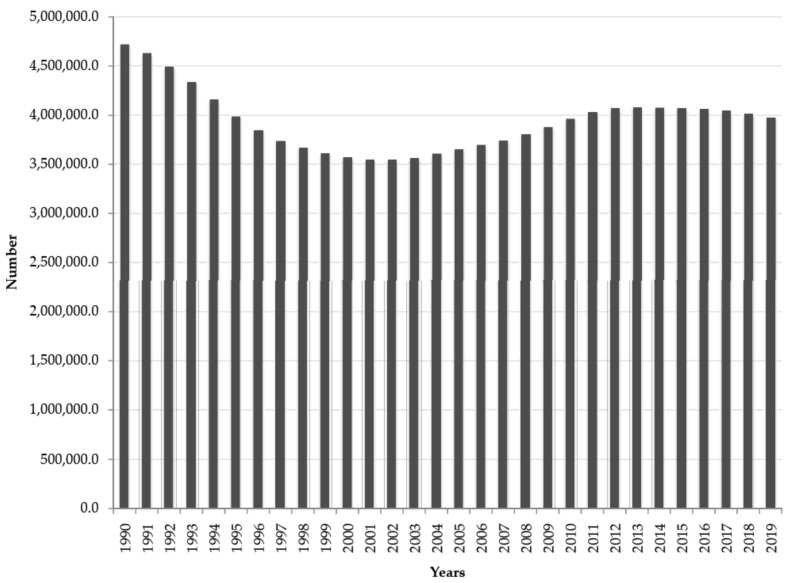
Atopic dermatitis in children under 5; prevalence in both sexes in the Central, Eastern, and Western European sub-regions, 1990–2019. Source: Global Burden of Disease estimates [8].

A total of 118 million (54 million males and 64 million females) prevalent cases of atopic dermatitis in children under 5 were reported in European countries in the 1990–2019 period (Figure 1). Annually, the number of cases ranged from 4.7 million in 1990 to 3.6 million in 2002 and then to 4.0 million in 2019.

**Figure 2:**
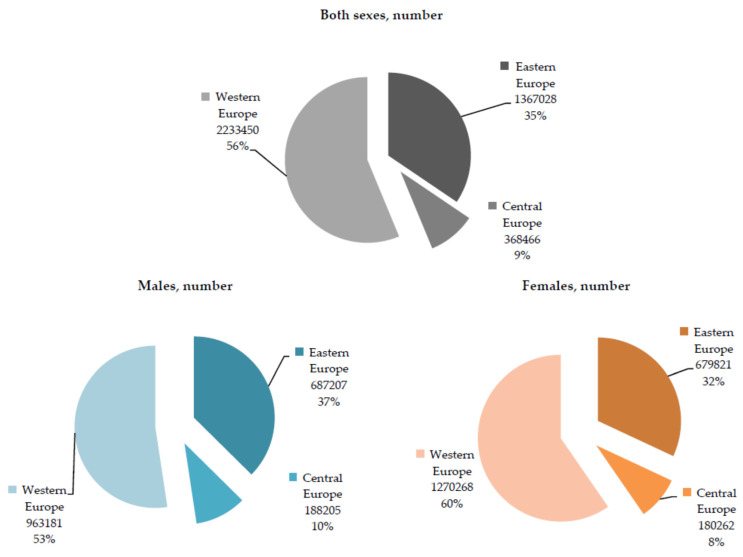
Atopic dermatitis in children under 5; prevalence in the Central, Eastern, and Western European sub-regions, by sex, in 2019. Source: Global Burden of Disease estimates [8].

More than half of all cases of atopic dermatitis in children of both sexes aged under 5 in Europe in 2019 were recorded in the Western European sub-region (56% of all cases in both sexes together; 2.2 million) (Figure 2). Similarly, males and females from the Western European sub-region made up the largest part of the total number of prevalent cases of atopic dermatitis in children aged under 5 in Europe (53% and 60%, respectively).

**Figure 3:**
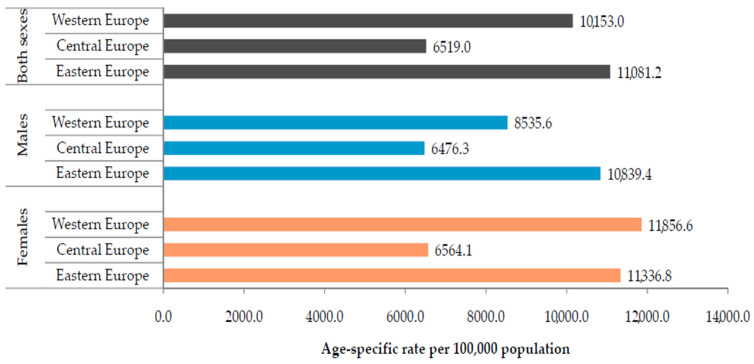
Atopic dermatitis in children under 5; age-specific rates of prevalence in the Central, Eastern, and Western European sub-regions, by sex, in 2019. Source: Global Burden of Disease estimates [8].

The prevalence rates of atopic dermatitis in children of both sexes aged under 5 were comparable in the Western and Eastern European sub-regions in 2019 (equally at over 10,000 per 100,000 population) (Figure 3). In both sexes, the prevalence rates were nearly two-fold lower than in the Central European sub-region. The highest prevalence rates were recorded in males in the Eastern European sub-region, while, in females, the highest prevalence rates were reported in the Western European sub-region.

**Figure 4:**
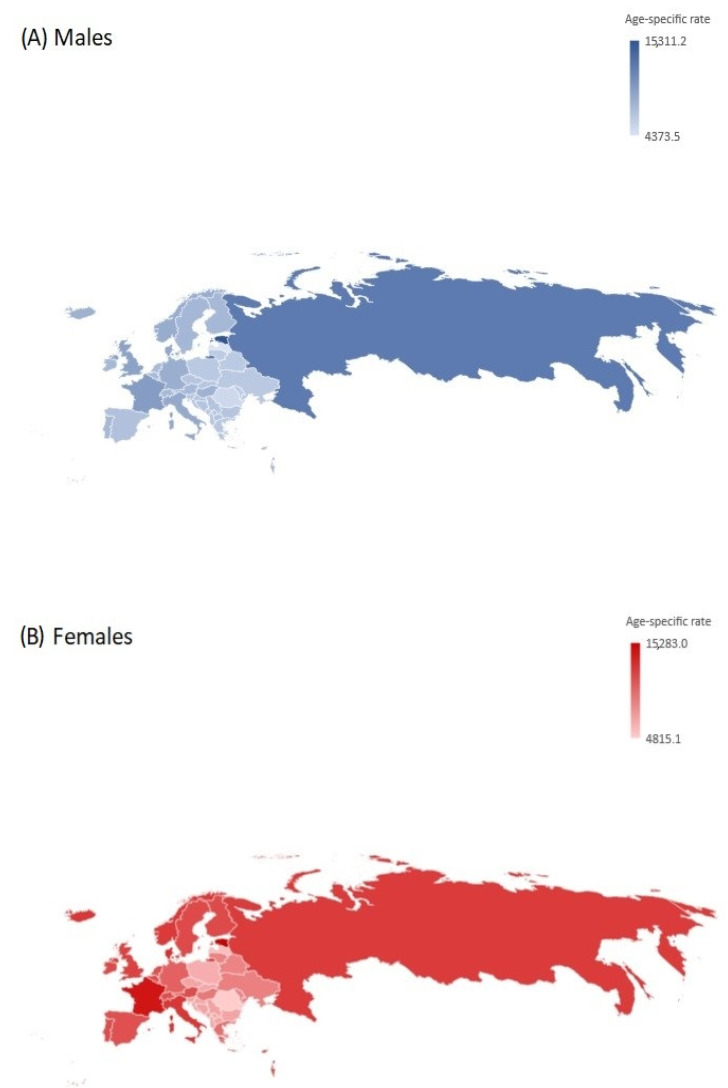
Atopic dermatitis in children under 5; age-specific rates (per 100,000) of prevalence in the Central, Eastern, and Western European sub-regions, by country and sex, in 1990–2019. Source: Global Burden of Disease estimates [8].

In almost all countries, the prevalence rates of atopic dermatitis in children under 5 were higher among females than males in 2019, with the exception of three countries in the Eastern European sub-region (Estonia, Romania, and Russian Federation) where slightly higher rates were reported in males (Figure 4, Table 1). The highest prevalence rates of atopic dermatitis in children aged under 5 were observed in the Eastern European sub-region, with the highest rates in both males and females recorded in Estonia (equally at about 15,000 per 100,000), followed by the Russian Federation (equally at about 12,000 per 100,000). A significantly increasing trend in atopic dermatitis prevalence in children under 5 was recorded in the Eastern European sub-region from 1990 to 2019 (AAPC = +0.3%, per year in males and +0.1% per year in females). The lowest prevalence rates were reported in Eastern Europe (equally at about 5000 per 100,000 in Romania and Latvia). Conversely, in the Western and Central European sub-regions, the trends of the prevalence of atopic dermatitis decreased (AAPC was about −0.1% per year).

**Figure 5:**
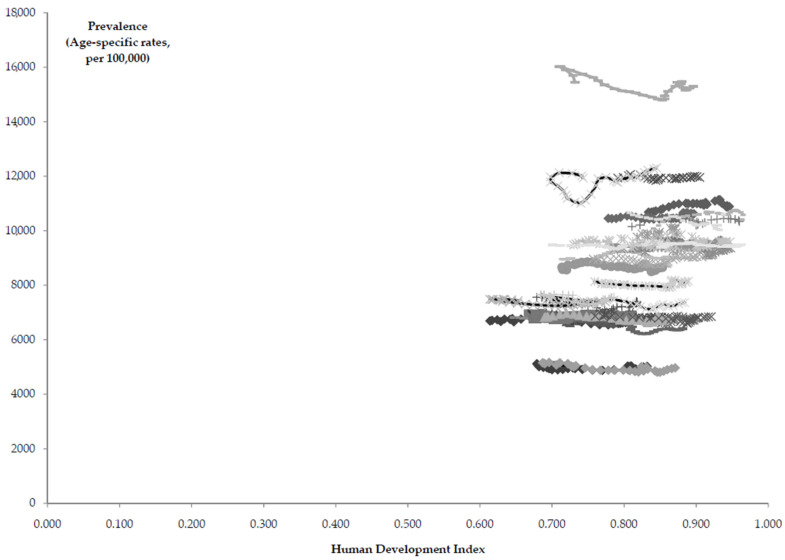
Atopic dermatitis in children under 5; correlation of the age-specific rates (per 100,000) in prevalence in the Central, Eastern, and Western European sub-regions with the Human Development Index, by country, in 1990–2019. Source: Global Burden of Disease estimates [8].

According to the Pearson coefficient, there was a significant negative correlation between the prevalence of atopic dermatitis in children under 5 and the HDI in most countries in the Eastern European sub-region (Table 1, Figure 5). A significant positive correlation was reported between the prevalence and HDI in high income countries in the Western European sub-region.

## 4. Discussion

The prevalence of atopic dermatitis in children under 5 significantly varied between the European countries. The largest prevalence rates in both sexes were observed in Estonia and the Russian Federation, while the lowest were reported in Romania and Latvia. The trends in atopic dermatitis in children under 5 in both sexes in many European countries were mostly stable. An opposite moderate correlation was observed in developed and developing countries between the prevalence of atopic dermatitis in children under 5 and social well-being as measured by the Human Development Index.

Atopic dermatitis represents a frequent chronic inflammatory skin disorder that presents with recurrent eczematous lesions and an intense itch, affecting up to 20% of children [5,17,18]. Previous studies have noted marked geographic variations in the prevalence of atopic dermatitis, with the highest rates observed in Sweden, the United Kingdom, and Iceland, as well as other Nordic countries, followed by North America [3,19,20]. Globally, the lowest prevalence rates of atopic dermatitis are reported in Uzbekistan, Armenia, and Tajikistan, as well as South Asia, Africa, and Latin America, which some authors attribute to under-registration and low industrialization and urbanization [19]. This study showed that the highest rates of prevalence of atopic dermatitis in children under 5 were observed in both sexes in Estonia and the Russian Federation in 2019, which could be due to the changes in the countries’ development level: all countries in the Eastern European region were part of the former Eastern European socialist bloc in the past; in the 1990s, they experienced different levels of social development and circumstances, whereby differences in the lag period of changes in society resulted in differences in the burden of diseases such as atopic dermatitis. On the other hand, low rates of atopic dermatitis in some countries in the Eastern European and Central European regions in part could be due to underreporting or the misdiagnosis of the disease in children [21,22].

Over the last few decades, in developed countries in North America (Canada and USA) and Europe, stable trends in the burden of disease for atopic dermatitis in children have been observed [3]. In this study, the rates of atopic dermatitis prevalence slightly decreased and were stable from 1990 to 2019 in almost all Western and Central European countries, while the prevalence rates of atopic dermatitis slightly increased in many Eastern European countries. Some authors have suggested that variations in the prevalence trends of atopic dermatitis in children under 5 could be related to differences across countries in urbanization, air pollution, water hardness, diet, microbiome alterations, psychosocial stress, parental smoking, the use of antibiotics, and cooking with firewood [23,24,25]. Moreover, some authors have suggested that changes in trends correlate with changes in environmental and/or intrinsic factors, such as poverty, ultraviolet exposure, climate change, nutritional status, and comorbidities (in particular, food allergies, asthma, allergic rhinitis) [19,25]. In addition, some reports point to the possible importance of early exposure to environmental factors, whereby etiological factors could act prenatally and earlier in the life course, causing an increase in the rate of atopic dermatitis in later life [20,23]. Moreover, a sex disparity in the prevalence and trends of atopic dermatitis in children under 5 was observed in this study: rates of atopic dermatitis prevalence decreased in both sexes in almost all Western and Central European countries, while the prevalence rates of atopic dermatitis increased in both sexes in many Eastern European countries. The reason for this difference is not entirely understood yet, while the effects of skin care practices, environmental exposure, and some unknown intrinsic factors could play a role in this phenomenon.

Similar to the results of this research, some studies indicate that the development of areas across the world is followed by changes in trends in the rates of prevalence of atopic dermatitis in children under 5, suggesting that economic prosperity is of great importance in the magnitude and direction of trends in atopic dermatitis [18,19]. With the prosperity of society comes better healthcare, the increased availability of improved preventive strategies, lifestyle changes, dietary habits, and tobacco control [23]. In this study, the Human Development Index was inversely correlated with the prevalence rates of atopic dermatitis in children under 5, suggesting that people in areas with a prosperous social economy and good well-being are more likely to have a healthy lifestyle and have access to higher levels of healthcare, which will be beneficial to the prevention and management of disease. Previous reports are consistent with our findings [18,26]. Some inconsistencies in the correlation between atopic dermatitis prevalence in children under 5 and the HDI can be linked to the fact that the HDI is only a surrogate measure for the development of a country, only suggesting a possible impact of lifestyle and other environmental factors (e.g., air pollution, use of antibiotics, hygiene measures, dietary factors, the skin and gut microbiomes) in the occurrence of disease.

The high level and trends of atopic dermatitis prevalence in children under 5 in the Central European sub-region in the last few decades constitute the magnitude of the disease burden in this area. Further efforts to harmonize the research methodology, explore the patterns of occurrence of atopic dermatitis, and identify risk factors implicated in this public health issue could be important for disease control. Finally, a comparison of the burden of disease for atopic dermatitis in children under 5 across countries should be carried out with caution, because the issue of underestimation or overestimation of the prevalence can always be raised, either due to the lack of a national database in most countries or to the issue of the largest number of relevant studies so far having been conducted in developed countries. Further efforts to provide data sources on atopic dermatitis prevalence and future studies are necessary to more accurately evaluate the trends of the disease and better understand the reasons for the changes in trends.

### Limitations of the Study

There are some sources of limitations in this study. Although the GBD estimates are updated every year, the quality of the data on which the estimates are based can always be questioned. Such concern always exists when estimates are based on sources derived from studies that apply different methodologies (some studies use the questionnaire method, while some studies use a standardized skin examination), and/or different definitions for the diagnosis of atopic dermatitis, as well as with a possible risk of misclassification of dermatitis [21,22]. A registry for atopic dermatitis on a global level, with the use of a standardized methodology and definitions, and with a uniform method of reporting, would significantly contribute to the more precise assessment of the disease burden, the evaluation of the undertaken measures of prevention and therapy, research into the etiology of the disease, and the development of more successful measures for the management of the disease. Further, the design of this study (i.e., descriptive/correlational study design) does not allow the evaluation of the relationship between the occurrence of atopic dermatitis and the Human Development Index in terms of potential causality, because data on individual exposures were not observed. Moreover, the lack of data on other characteristics of cases (such as lifestyle factors and environmental exposure, including socio-economic status, air pollution, dietary habits, hereditary factors, comorbidities) is a drawback of this study. Finally, due to the delay in obtaining data during the COVID-19 pandemic, cases of atopic dermatitis in children under 5 for the last few years could not be included in this analysis.

## 5. Conclusions

Across European countries, large differences exist in the prevalence of atopic dermatitis in children under 5. The highest prevalence rates and slightly increasing trends of atopic dermatitis were seen in both sexes in Eastern European countries from 1990 to 2019. Moreover, despite the mainly stable trends observed in both sexes in most developed European countries, atopic dermatitis shows a high burden in many countries. Future studies should estimate which factors contribute to the large geographic differences in the prevalence of atopic dermatitis in children under 5, which could facilitate the identification of more effective disease control measures.

## Figures and Tables

**Table 1:** Atopic dermatitis in children under 5; age-specific rates (per 100,000) of prevalence in the Central, Eastern, and Western European sub-regions, by location and sex, in 1990–2019; a Joinpoint regression analysis * and correlation with Human Development Index (HDI).

Locations **	Males			Females			Both Sexes—HDI	
	1990	2019	AAPC	1990	2019	AAPC	Pearson Coefficient	*p*
Western Europe	8497.4	8535.6	−0.1 *	11,768.9	11,856.6	0.0	-	-
Central Europe	6473.9	6476.3	−0.1 *	6577.8	6564.1	−0.1 *	-	-
Eastern Europe	10,116.0	10,839.4	0.3 *	10,980.6	11,336.8	0.1 *	-	-
Albania	6576.5	6538.6	−0.1 *	6739.6	6733.7	−0.1 *	−0.628	0.000
Andorra	7742.6	7893.9	+0.2 *	11,556.9	11,754.3	0.1 *	0.632	0.003
Austria	7599.5	7635.3	−0.0	11,324.5	11,374.6	−0.0	−0.239	0.204
Belarus	6379.7	6325.7	−0.2 *	8626.4	8544.4	−0.1 *	−0.621	0.001
Belgium	7569.6	7653.5	0.0	11,276.0	11,404.4	0.0	0.234	0.214
Bosnia and Herzegovina	6743.5	6784.3	−0.0	6947.2	6991.7	−0.0	−0.354	0.126
Bulgaria	6754.8	6719.0	−0.1 *	6963.7	6923.0	−0.1 *	−0.514	0.004
Croatia	6759.0	6716.7	−0.0	6967.5	6923.4	−0.0	−0.375	0.041
Cyprus	7029.1	7093.0	−0.0	10,997.1	11,099.0	−0.0	−0.251	0.181
Czechia	6683.9	6695.6	−0.1 *	6889.0	6900.6	−0.1 *	−0.493	0.006
Denmark	8832.4	9002.7	0.0 *	12,626.1	12,872.1	0.1 *	0.700	0.000
Estonia	15,448.3	15,311.2	−0.1 *	15,439.4	15,283.0	−0.2 *	−0.698	0.000
Finland	7555.6	7717.7	0.0 *	11,253.7	11,496.8	0.0*	0.418	0.021
France	9806.7	9815.9	−0.0	14,168.4	14,181.7	0.0	−0.093	0.626
Germany	8354.4	8480.5	−0.0	10,164.3	10,298.4	−0.0	−0.344	0.063
Greece	6666.3	6655.0	0.0	9682.7	9711.0	0.0	0.042	0.825
Hungary	8092.0	8180.7	−0.0 *	9052.4	9149.5	−0.1 *	−0.408	0.025
Iceland	8139.4	8258.9	−0.0	12,246.9	12,466.6	0.0	0.003	0.989
Ireland	7764.1	7709.1	−0.0	11,580.6	11,486.1	−0.0	−0.289	0.122
Israel	7647.0	7587.7	0.0 *	11,399.5	11,306.8	−0.0 *	−0.591	0.001
Italy	8688.0	8832.1	0.1 *	12,313.2	12,512.9	0.1 *	0.520	0.003
Latvia	4394.2	4373.5	−0.2 *	5628.8	5591.7	−0.2 *	−0.770	0.000
Lithuania	6258.4	6289.8	−0.1 *	8459.2	8498.9	−0.1 *	−0.734	0.000
Luxembourg	7516.4	7629.2	0.1 *	11,189.9	11,371.8	0.1 *	0.771	0.000
Malta	7653.3	7658.8	−0.1 *	11,402.1	11,386.8	−0.1 *	−0.703	0.000
Monaco	7376.2	7700.2	0.1 *	10,865.2	11,416.9	0.1 *	-	-
Montenegro	6749.3	6710.5	−0.0 *	6962.1	6909.9	−0.0 *	−0.033	0.901
Netherlands	7554.7	7610.9	0.0 *	11,258.8	11,340.1	0.0 *	0.471	0.009
North Macedonia	6683.7	6694.9	−0.0 *	6898.9	6900.5	−0.1 *	−0.598	0.002
Norway	8344.0	8611.8	0.1 *	11,897.5	12,283.7	0.1 *	0.760	0.000
Poland	6625.3	6409.3	−0.2 *	6708.1	6391.5	−0.3 *	−0.790	0.000
Portugal	7650.2	7679.5	0.0 *	11,395.7	11,442.0	0.0 *	0.409	0.025
Republic of Moldova	6335.1	6310.9	−0.1 *	8568.6	8527.0	−0.1 *	−0.561	0.001
Romania	5243.9	5213.8	−0.0	4814.7	4815.1	−0.0	−0.180	0.342
Russian Federation	11,819.1	12,350.6	0.2 *	12,038.0	12,248.7	0.1	0.542	0.002
San Marino	7683.1	7683.2	0.0 *	11,486.2	11,458.9	0.0 *	-	-
Serbia	6564.6	6800.7	0.1 *	6936.2	6997.2	0.0	0.453	0.012
Slovakia	6720.9	6678.0	−0.1 *	6928.8	6881.7	−0.1 *	−0.684	0.000
Slovenia	6794.2	6746.0	−0.1 *	7007.8	6949.5	−0.1 *	−0.610	0.000
Spain	6963.1	7016.6	0.1 *	11,096.4	11,173.8	0.0	0.260	0.165
Sweden	7565.0	7751.3	−0.0	11,263.6	11,539.0	−0.0	−0.055	0.772
Switzerland	7575.9	7657.9	−0.0	11,291.6	11,407.3	−0.0	−0.350	0.058
Ukraine	6490.5	6446.3	−0.1 *	8807.1	8734.6	−0.1 *	0.571	0.001
United Kingdom	9417.4	9336.6	−0.1 *	11,965.9	11,921.7	−0.1 *	−0.788	0.000

* Statistically significant trend (*p* < 0.05). ** Results of correlation analysis are not presented for prevalence in certain locations, due to the absence of data regarding Human Development Index in the observed period. AAPC = For full period, presented Average Annual Percent Change. Source: Global Burden of Disease estimates [8].

## Data Availability

Data are contained within the article.
